# Chemokine–receptor-guided B-cell immunity in cardiovascular disease

**DOI:** 10.1007/s00395-025-01140-x

**Published:** 2025-09-23

**Authors:** Anais Yerly, Emiel P. C. van der Vorst, Marc Schindewolf, Drosos Kotelis, Heidi Noels, Yvonne Döring

**Affiliations:** 1 Division of Angiology, Swiss Cardiovascular Center, Inselspital, Bern University Hospital, University of Bern, Freiburgstrasse, CH-3010 Bern, Switzerland; 2 Department for BioMedical Research (DBMR), University of Bern, Bern, Switzerland; 3 Graduate School for Cellular and Biomedical Sciences, University of Bern, Bern, Switzerland; 4 Institute for Molecular Cardiovascular Research (IMCAR), RWTH Aachen University, 52074 Aachen, Germany; 5 Aachen-Maastricht Institute for CardioRenal Disease (AMICARE), RWTH Aachen University, 52074 Aachen, Germany; 6 Department of Internal Medicine I, University Hospital Aachen, RWTH Aachen University, Aachen, Germany; 7 Institute for Cardiovascular Prevention (IPEK), Ludwig-Maximilians-University Munich (LMU), 80336 Munich, Germany; 8 Department of Vascular Surgery, Inselspital, Bern University Hospital, University of Bern, Bern, Switzerland; 9 Department of Biochemistry, Cardiovascular Research Institute Maastricht (CARIM), Maastricht University, Maastricht, the Netherlands; 10 DZHK (German Centre for Cardiovascular Research), Partner Site Munich Heart Alliance, 80336 Munich, Germany

**Keywords:** B cells, Cardiovascular diseases (CVD), Atherosclerosis, Chemokines, Atypical chemokine receptors, Inflammation

## Abstract

Cardiovascular diseases (CVD) include a wide range of disorders affecting the heart and blood vessels, many of which are associated with atherosclerosis. Atherosclerosis is the main underlying cause of CVDs and represents a chronic inflammatory disease of the large arteries involving the build-up of plaques within the arterial wall. B cells play a dual role in CVD, particularly in the context of atherosclerosis, by producing antibodies and secreting cytokines that modulate inflammation. Depending on their subtype (B1 vs. B2 cells) and the specific context, B cells can have both protective and harmful effects on the cardiovascular system. B1 cells, which arise predominantly during fetal development, are found in body cavities, such as the perivascular adipose tissue (PVAT) and peritoneum. Guided by CXCL13 and CCR6, they migrate to sites, where they produce IgM and IgG3, contributing to immune regulation and pathogen defense. In contrast, B2 cells—central players in adaptive immunity—originate in the bone marrow and mature in secondary lymphoid organs. Within this subset, marginal-zone (MZ) B cells provide rapid, low-affinity IgM responses to blood-borne antigens, while follicular (FO) B cells mediate high-affinity, T-cell-dependent antibody production. For all of the latter chemokine-guided migration is essential for B-cell function, from immune surveillance to antibody secretion. Receptors such as CXCR4, CXCR5, and ACKR3 not only direct B-cell trafficking but also influence their phenotype in cardiovascular disease. Understanding how these chemokine–receptor interactions shape B-cell-mediated immunity in CVD may allow for developing targeted therapies for atherosclerosis, myocardial infarction, and stroke.

## Introduction

Cardiovascular diseases (CVD) are the leading cause of death worldwide, accounting for an estimated 17.9 million deaths annually (WHO). They encompass disorders, such as coronary artery disease (CAD), peripheral artery disease (PAD), stroke, heart failure, and arrhythmia, many of which share a common pathological basis in atherosclerosis. Beyond local vascular inflammation, extra-cardiac immune reservoirs such as the spleen are now recognized as critical modulators of ischemic heart disease, influencing both onset and progression [53]. While classic risk factors such as hypertension, dyslipidaemia, diabetes mellitus, and smoking contribute substantially to disease burden, emerging evidence highlights the role of chronic inflammation, immune dysregulation, psychosocial stress, and certain infections in CVD pathogenesis [40, 106]. Inflammation in cardiometabolic comorbidities (diabetes, hypertension) also affects costimulatory axes, such as CD40–CD40L–TRAF, offering non-chemokine immunomodulatory entry points that may interface or synergize with B-cell activation [104]. Beyond inflammation per se, *immunometabolic* reprogramming of immune and stromal cells is increasingly recognized as a driver of cardiovascular pathology—particularly during the transition from ischemic injury to heart failure—providing a unifying framework in which chemokine-guided leukocyte behavior matters significantly [5].

Atherosclerosis, the principal underlying cause of many CVDs, is a chronic inflammatory disease of the large arteries characterized by plaque formation within the arterial wall. Endothelial injury increases vascular permeability, allowing circulating lipids to infiltrate the intima, where they undergo oxidation, triggering an inflammatory response and recruitment of immune cells, particularly monocytes. These differentiate into macrophages that engulf oxidized lipids and become foam cells [46]. Smooth muscle cells migrate from the media to the intima, proliferate, and secrete extracellular matrix, forming the fibrous cap [43]. Other immune cells, such as T cells, neutrophils, and mast cells, are also recruited to the intima, where they contribute to the inflammation process [88]. Interactions between immune cells, inflammatory mediators, and vascular tissues drive the progression of atherosclerotic plaques and can lead to acute cardiovascular events. Chemokines, a subset of chemotactic cytokines, and their receptors are central to orchestrating immune-cell recruitment and activation within the cardiovascular system [30], making them key players in atherosclerosis and other CVDs [41, 112].

B cells exert both protective and pathogenic roles in atherosclerosis through antibody production and cytokine secretion. Atheroprotective effects have been ascribed to B1 cells, marginal-zone B2 cells, and regulatory B cells (Bregs)—the latter arising from both B1 and B2 lineages and capable of attenuating inflammation and stabilizing plaques [68, 69, 80, 93]. In contrast, other B2 subsets can promote inflammation and drive plaque progression [68, 69, 80, 93]. Hence, the diverse roles of B cells in CVD may be essential for developing targeted therapies to modulate their activity, reduce disease progression, and improve cardiovascular health.

This review focuses on chemokine-(receptor)-driven B-cell immunity in CVD with emphasize on the CXCR4/CXCL12/ACKR3 chemokine/receptor axis.

## Chemokines and their (atypical) receptors

Chemokines are chemotactic cytokines which form a large family of small secreted proteins (8–12 kDa) that are best known for their ability to induce chemotaxis of immune cells [49, 77]. To date, more than 50 chemokines have been identified. They are divided into four subfamilies based on their two last cysteine residues of the N terminus, giving them a distinct three-dimensional shape: currently, the chemokine system includes 28 CCs, 16 CXCs, 1 CX_3_C, and 2 XCs chemokines [57]. The classical, also called conventional, chemokine receptors are 7-transmembrane (7TM) receptors. At least 18 classical chemokine receptors have been identified: 10 CCRs, 6 CXCRs, 1 CX_3_CR, and 1 XC [57], and many of those can bind to more than one receptor and vice versa [57]. Upon ligand binding, the chemokine receptor will undergo conformational changes, which enable the binding to G-proteins. The activated G protein initiates an intracellular signaling cascade that regulates the activation of transcription factors and subsequently gene expression. Chemokines and their receptors are involved both in homeostasis and inflammatory conditions, and, therefore, exhibit a large spectrum of characteristics and functions [49]. They can influence many cellular and physiological processes, such as cell mobilization, migration, survival, proliferation, differentiation, activation of other cytokines, positioning of immune cells, but also developmental processes, such as haematopoiesis, angiogenesis, and neurogenesis, as well as autoimmune reactions [57, 94]. Atypical chemokine receptors (ACKRs) are 7TM receptors of which the second intracellular loop lacks a specific amino acid sequence, the canonical Asp–Arg–Tyr–Leu–Ala–Ile–Val (DRYLAIV) motif, which is responsible for G-protein binding. As a consequence, they cannot induce G-protein coupled intracellular signaling leading to chemotaxis [13, 85]. Instead, ACKRs are considered decoy receptors. Upon binding of their cognate ligands, ACKRs can internalize, scavenge, transport, or present ligands to other cells [44]. Therefore, they indirectly control the interactions of the chemokine ligands with their conventional chemokine receptors by modulating chemokine abundance, distribution, and localisation [57]. ACKRs comprise four chemokine receptors, which include ACKR1 (previously known as DARC), ACKR2 (previously known as D6 or CCBP2), ACKR3 (previously known as CXCR7 or RDC1), and ACKR4 (previously known as CCRL-1 or CCX–CKR). In addition, GPR182 (CCRL2) and PITPNM3 may be joining the ACKR family as ACKR5 and ACKR6, respectively. These two receptors are still under investigation and await functional confirmation [13, 14]. Overall, chemokines can bind to both classical and atypical chemokine receptors, and the predominant interaction between ligand and receptor is determined, for example, by receptor expression patterns, binding affinities, post-translational modifications, and microenvironmental conditions [14].

## Chemokine–(receptors) orchestrate B-cell positioning and fate

B-cell traffic between bone marrow, secondary lymphoid organs, and tissue niches is orchestrated by a small set of chemokine axes that determine, where B cells develop, examine antigen, and secrete antibodies. Among these, CXCL12–CXCR4 and CXCL13–CXCR5 partition germinal-center dark/light zones and guide bone-marrow homing of plasmablasts and long-lived plasma cells; ACKR3 reshapes CXCL12 gradients and tunes subset positioning. We next outline how these cues act on the main B-cell lineages (B1 and B2) and their subsets in CVD [30, 43, 71, 93].

### B1 cells

B cells comprise two major families: B1 cells and B2 cells (“conventional” B cells). The developmental origin of B1 cells remains debated—whether they arise from distinct progenitors independent of the classical hematopoietic stem cell (HSC) pathway or share a precursor with B2 cells is still unresolved [76]. However, it is known that B1 cells emerge predominantly from the fetal liver during prenatal to early postnatal life and expand through homeostatic proliferation [35]. In adults, they may also derive in part from bone-marrow (BM) progenitors distinct from those generating B2 cells, although this contribution is minor compared to fetal-derived BM [8, 35, 76]. B1 cells can be further divided into B1a cells, which are primarily involved in natural antibody production, and B1b cells, which contribute mainly to adaptive, antigen-induced immune responses [4, 24, 76].

**Fig. 1:**
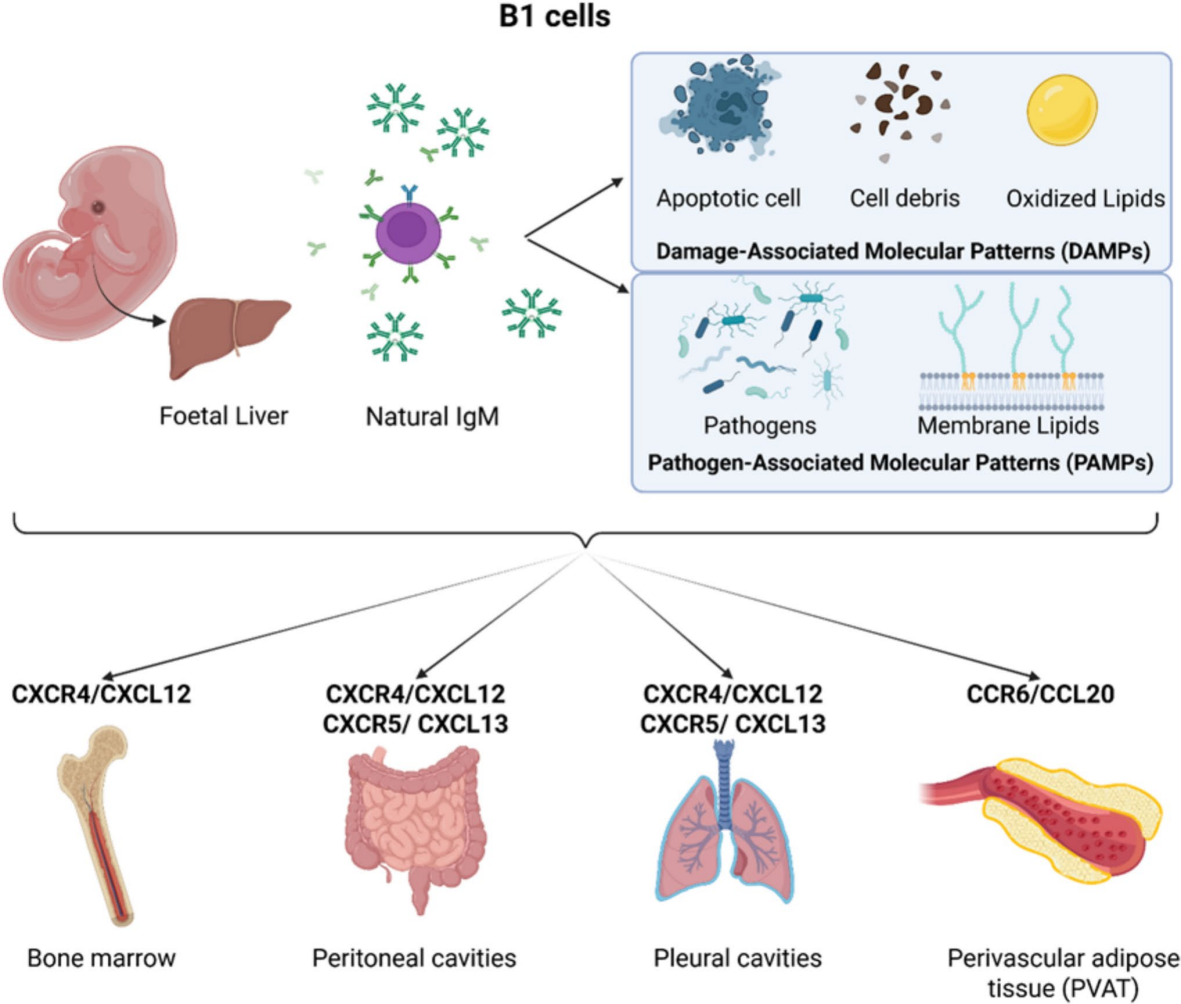
Chemokine (receptor)-mediated regulation of B1 cell tissue localization. B1 cells originate mainly from the fetal liver and are known for producing natural IgM, which plays a crucial role in early immune defense mechanisms. The IgM antibodies produced by B1 cells target various molecular patterns, which are categorized into two types: i) damage-associated molecular patterns (DAMPs), which include apoptotic cells (dying cells), cell debris, and oxidized lipids and are, therefore, endogenous molecules that signal tissue damage and ii) pathogen-associated molecular patterns (PAMPs), which include pathogens like bacteria, highly conserved pattern recognition receptors (PRRs) such as toll-like receptors (TLRs), and pathogen-associated membrane lipids and are, therefore, recognized as foreign by the immune system, prompting a response. B1 cells migrate to and are found in various anatomical sites, facilitated by specific chemokine–chemokine receptor interactions: B1 cells are recruited to perivascular adipose tissue (PVAT) via the CCR6/CCL20 signaling pathway, while recruitment and migration to the bone marrow, pleural & peritoneal cavities are mediated by the CXCR4/CXCL12 and CXCR5/CXCL13 signaling axis. The figure was created in BioRender.com

B1 cells are enriched in the pleural and peritoneal cavities but also localize to perivascular adipose tissue (PVAT), BM, spleen, and lymph nodes [76]. Moreover, the spleen, in particular, is a central reservoir and regulator of immune cells in ischemic heart disease, also influencing B-cell positioning and function [53]. Their positioning is shaped by chemokine axis. CXCR4–CXCL12 signaling directs their homing to the BM and peritoneal and pleural compartments, where they expand and differentiate into mature B1 cells (Fig. 1) [79, 108]. Furthermore, egress of B1 cells out of the peritoneal cavity and homing in body cavities is also dependent on chemokine receptor CXCR5 and its ligand CXCL13, which is expressed by local macrophage populations. At the same time, their migration to PVAT is mediated via CCR6 and its ligand CCL20 (Fig. 1) [6, 54, 102]. During B-cell development in the BM, CCR6 is initially absent but is expressed on mature B cells in peripheral blood and other tissues. Mature B cells lose CCR6 expression upon activation through B-cell receptor signaling but re-express it when differentiating into memory B cells [102].

Mature B1 cells recognize, bind, and respond to conserved epitopes from damage- and pathogen-associated molecular patterns (DAMPs and PAMPs) [100] and are the are major producers of “natural” antibodies—primarily IgM and, to a lesser extent, IgG3—that are generated without prior antigen exposure (Fig. 1). These antibodies regulate immunity, suppress autoantibody formation, and defend against pathogens. IgM production is driven mainly by B1 cells in the BM and spleen [21]. In PVAT, locally produced IgM by B1 cells modulates inflammation by interacting with macrophages and facilitating the clearance of apoptotic cells [102] B1 cells can be divided into Blimp-1-dependent and Blimp-1-independent subsets [96]. Classical B1 cells and B1-derived plasma cells require Blimp-1 for antibody secretion, but certain BM-resident B1 cells secrete IgM independently of Blimp-1. Similarly, IgG3 production by B1 cells is largely Blimp-1-independent, maintaining serum IgG3 even in the absence of Blimp-1-dependent secretion [96].

Through their chemokine-directed positioning and specialized antibody repertoire, B1 cells contribute to immune surveillance, tissue homeostasis, and the clearance of oxidized lipids implicated in atherosclerosis.

### B2 cells

B2 cells originate in the BM, where precursor cells acquire a unique B-cell receptor (BCR) composed of immunoglobulin (Ig) heavy and light chains through V(D)J recombination [110]. Immature B cells initially express IgM on their surface. Differentiation from hematopoietic stem cells (HSCs) into pro-B cells requires close contact with CXCL12-expressing stromal cells, while subsequent maturation depends on signals from IL-7-producing stromal cells [107]. Retention of B-cell precursors in the BM is mediated by CXCL12–CXCR4; in CXCR4-deficient mice, immature B cells exit prematurely, accumulating in splenic follicles, while mature B cells are reduced in the splenic follicle (FO), marginal zone (MZ), and peritoneal cavity [79]. These mice also showed increased immature B cells in the splenic follicle. At the same time, mature B cells were decreased in the splenic FO but also in the MZ, primary follicle (precursor state of the germinal center), and peritoneal cavity [79]. Successful heavy-chain rearrangement generates pre-B cells with a light chain forming the pre-BCR. Light-chain rearrangement produces a complete BCR, allowing progression to the immature stage. Reduced CXCR4 expression enables immature B cells to leave the BM and migrate to secondary lymphoid organs, such as the spleen and LN [49]. Homing to these sites is mediated by CCR7–CCL21 and CXCR5–CXCL13 gradients, attracting CCR7⁺CXCR5⁺ cells to high endothelial venules (via CCL21) and follicular dendritic cell (FDC) or T follicular helper (Tfh)-derived CXCL13 [82].

Within the spleen, B2 cells populate distinct compartments. The red pulp filters blood, while the white pulp contains T-cell-rich periarteriolar lymphoid sheaths (PALS) and adjacent B-cell follicles. The latter is surrounded by the MZ, which is rich in macrophages, dendritic cells (DCs), and MZ B cells [73]. Positioned between red and white pulp, MZ B cells rapidly respond to blood-borne antigens by producing large quantities of low-affinity IgM antibodies [20]. Notably, in humans, a B-cell subset with characteristics similar to those of circulatory MZ B cells has been identified as an important producer of atheroprotective IgM [86]. Furthermore, recent evidence highlights that splenic organization not only orchestrates these compartments but also has systemic relevance in ischemic heart disease, underlining the spleen’s role as an immune hub [53].

**Fig. 2:**
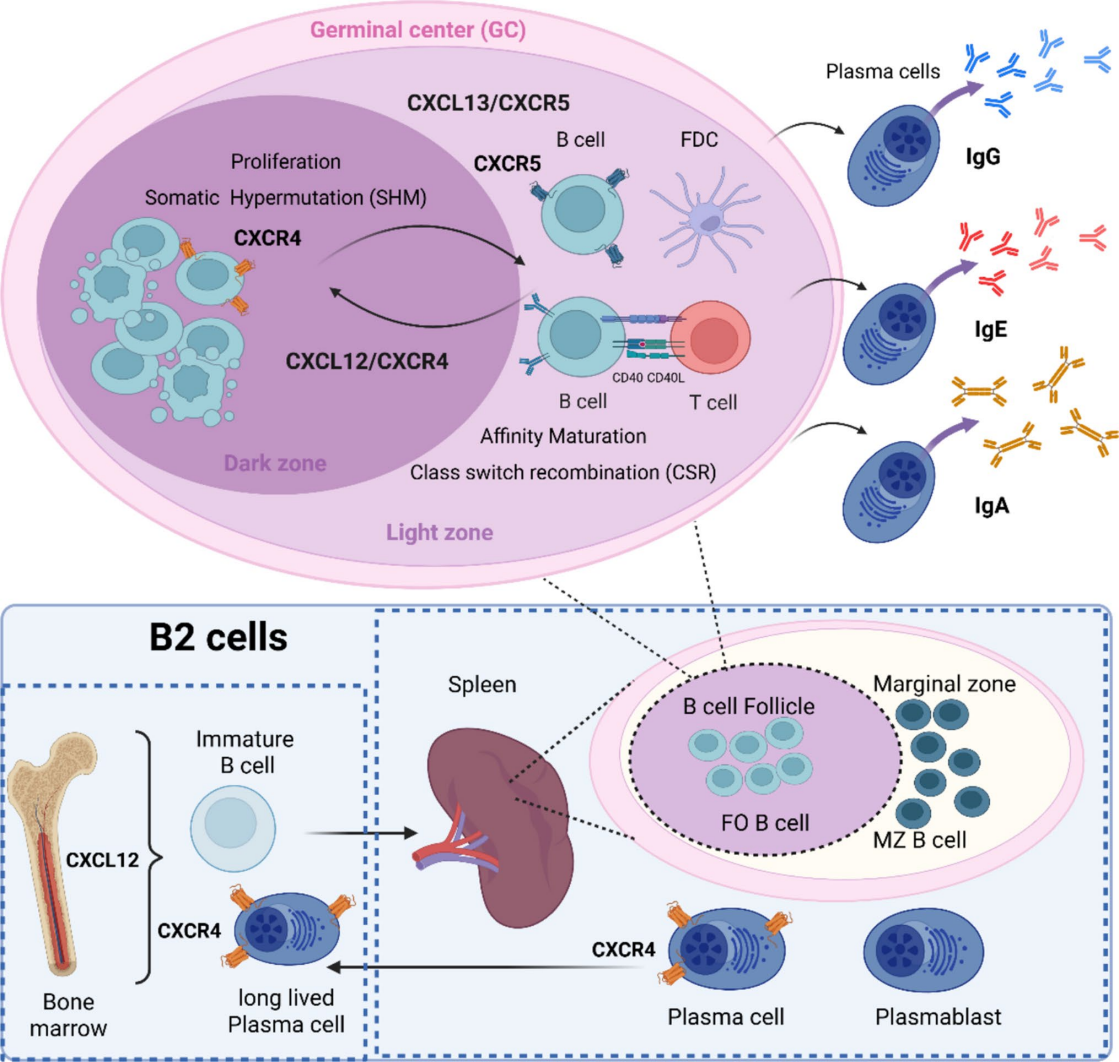
Chemokine (receptor)-mediated B2 cell immune response. Immature B2 cells originating from the bone marrow (BM) migrate to secondary lymphoid organs (SLO) for further maturation. In the spleen, the immature B cell can either become a marginal-zone (MZ) B cell or a follicular zone (FO) B cell. Positioned between white and red pulp, MZ B cells respond to blood-borne antigens by producing IgM antibodies and can be activated in a T-cell-independent manner. Located in the B-cell follicle, FO B cells are involved in T-cell-dependent antibody responses. Upon antigen encounter, they migrate in the direction of the T cell zone adjacent to the FO via CCL19/CCL21/CCR7 and undergo class-switch recombination (CSR) in the germinal center (GC), leading to the production of various antibody isotypes. In the dark zone of the GC, B cells proliferate and undergo somatic hypermutation (SHM) influenced by CXCL12/CXCR4 migration. In the light zone, B cells undergo affinity maturation and CSR facilitated by CXCL13/CXCR5 signaling. Interactions between follicular dendritic cells (FDCs) and T cells drive these processes through the interaction of CD40 and CD40L. Selected B cells then differentiate into plasma cells, producing high-affinity IgG, IgE, or IgA. After this maturation process, B cells become highly specific antibody-producing plasma cells. The plasma cells leave the spleen and upregulate CXCR4 surface receptors, which enable them to home to the BM by following a CXCL12 chemokine gradient. The figure was created in BioRender.com

B2 cells express polyreactive BCRs, complement receptors (CD21, CD35, CD1d), and toll-like receptors (TLRs) [9, 22]. Their shuttling between the MZ sinus and white pulp is regulated by sphingosine-1-phosphate (S1P)–S1P₁ gradients [66], and CXCR5–CXCL13 signaling. Upon antigen encounter, downregulation of S1P₁ and upregulation of CXCR5 direct migration into follicles to interact with FDCs and T cells.[22] FO B cells reside in splenic follicles and mount T-cell-dependent antibody responses (Fig. 2). Antigen recognition—directly or via DC/macrophage presentation—induces CCR7 upregulation, guiding FO B cells towards CCL19/CCL21-rich T-cell zones [66, 78]. Co-stimulation via CD40–CD40L interactions with activated T helper cells, along with cytokine signals, drives germinal-center (GC) formation and class-switch recombination to IgG, IgA, or IgE (Fig. 2) [34]. The fully activated FO B cells will begin to form the GC and become GC B cells. Within the GC, CXCL12–CXCR4 and CXCL13–CXCR5 gradients maintain dark and light zone segregation [26] CXCR4 upregulation directs GC B cells to the dark zone for proliferation and somatic hypermutation (SHM), while CXCR5 upregulation returns them to the light zone for affinity-based selection. Class-switch recombination of IgG, IgA, or IgE isotypes in the light zone is mediated by activation-induced cytidine deaminase [26]. High-affinity clones differentiate into memory B cells or plasma cells [78]. After leaving the spleen, memory B cells and plasma cells re-enter circulation via S1P/S1P₁ and home to the BM under the guidance of CXCL12–CXCR4. Plasma cells upregulate CXCR4 while downregulating CCR7 and CXCR5 to facilitate BM retention [62].

## Chemokine (receptor) guided B-cell functions in myocardial infarction and stroke

Myocardial infarction (MI), also known as a heart attack, is a critical medical condition marked by the abrupt cessation of blood flow to a segment of the heart muscle [38]. After MI, necrotic myocytes release DAMPs, cytokines, and autoantigens, which trigger an inflammatory cascade that prompts the rapid influx of immune cells into the infarcted area [48]. B cells have a dual role in MI, contributing to both inflammation and tissue repair. Mature B cells produce chemokines like CCL7, recruiting Ly6C^high^ monocytes to the infarcted myocardium and worsening left ventricular ejection fraction [115]. They also interact with CD4 + T cells, triggering the release of pro-inflammatory cytokines. Conversely, CD11b-B1 cells secrete IL-4, IL-10, and IgM, promoting anti-inflammatory effects and tissue repair, which improves ventricular function post-MI [18].

Bregs, on the other hand, improve ventricular remodeling by modulating monocyte infiltration in the heart. Mechanistically, Bregs exert their protective effects by secreting IL-10, which downregulates CCR2 expression in monocytes, thereby inhibiting the recruitment of pro-inflammatory monocytes from peripheral blood and their mobilization from the BM into the heart [61]. Bregs were also investigated in myocardial ischemia–reperfusion injury (MI/RI). Following MI/RI, rapid recruitment of B cells and Bregs to the heart was observed, exerting significant immunomodulatory effects [56]. Despite their low abundance compared to neutrophils, B cells are strategically localized in the microvasculature, facilitating direct interactions with infiltrating neutrophils and enhancing their impact on inflammation resolution [56]. In addition, a rapid influx of polyclonal B cells into the myocardial scar tissue post-MI was observed, accompanied by a modest expansion of oligoclonal GC B cells in LNs. Specifically, a CXCR5 + CCR7 + B-cell subset was identified that migrates to the heart via the CXCL13 axis, contributing to local TGF-ß1 production, which is crucial for myocardial healing. Neutralization of CXCL13 using specific antibodies, combined with the use of CXCR5-deficient mice, resulted in a decreased infiltration of B cells and reduced local TGF-ß1 expression post-MI. However, no differences in contractile function or myocardial morphology were observed between the experimental groups [52]. In conclusion, compensatory cytokine activity, redundancy in cellular roles during healing, and possible contributions from other sources of TGF-β1 likely allowed the myocardium to maintain contractile function despite reduced B-cell-mediated TGF-β1 production. This suggests that while CXCR5 + CCR7 + B cells and TGF-β1 contribute to the post-MI healing process, they are not solely responsible for preserving cardiac structure or function in this context. Post-MI inflammation and repair unfold alongside profound shifts in immune-cell metabolism. Integrating these immunometabolic programs with chemokine cues (for example, CXCL12–CXCR4–ACKR3) may help explain why similar cellular infiltrates can yield divergent remodeling outcomes and heart-failure trajectories [5]. Humoral responses also modulate post-MI remodeling. Exposure of cardiac autoantigens after necrosis can elicit B-cell activation and antibody production, while B-2-derived chemokines (e.g., CCL7) recruit inflammatory monocytes that worsen function. Conversely, natural IgM and IL-10-producing B-cell subsets restrain inflammation, enhance clearance of apoptotic debris, and improve ventricular remodeling. These data position antibody-secreting B cells as bidirectional modulators of infarct healing [56, 61, 115].

In stroke recovery, B cells have emerged as pivotal players due to their role in antibody production and antigen presentation within the adaptive immune system [12]. B-cell infiltration into the brain occurs via CXCL13/CXCR5 interaction. Following a stroke, there is a marked influx of B cells into the brain, particularly in regions, such as the cortex and subcortex, where increased levels of the chemokine CXCL13 are found. This migration extends to regions distant from the stroke site, suggesting a broad impact on neuroinflammatory processes [75, 83]. Research indicates that B cells exert neuroprotective effects by secreting neurotrophic factors, including brain-derived neurotrophic factor (BDNF), nerve growth factor (NGF), and IL-10 [33, 36, 64]. The timing and nature of B-cell activation following stroke are critical factors influencing their role in stroke pathology and repair. Early studies suggest that B cells may have limited influence during the acute phase (first 1–3 days) of stroke but become increasingly significant during the subacute and chronic phases [83, 98]. Moreover, antibody-secreting B cells are detected in peri-infarct niches after stroke. Here, natural IgM may neutralize DAMPs and foster debris clearance, whereas timing and context determine whether humoral responses are protective or neutral in the acute phase. In models, IL-10-competent B cells and B-cell-derived trophic factors support recovery, highlighting therapeutic windows in the subacute/chronic stages [12, 83, 98]. Hence, the ability of B cells to modulate neuroinflammation and promote neurogenesis underscores their potential as therapeutic targets for enhancing stroke recovery.

## Chemokine (receptor) guided B-cell functions in atherosclerosis

**Fig. 3:**
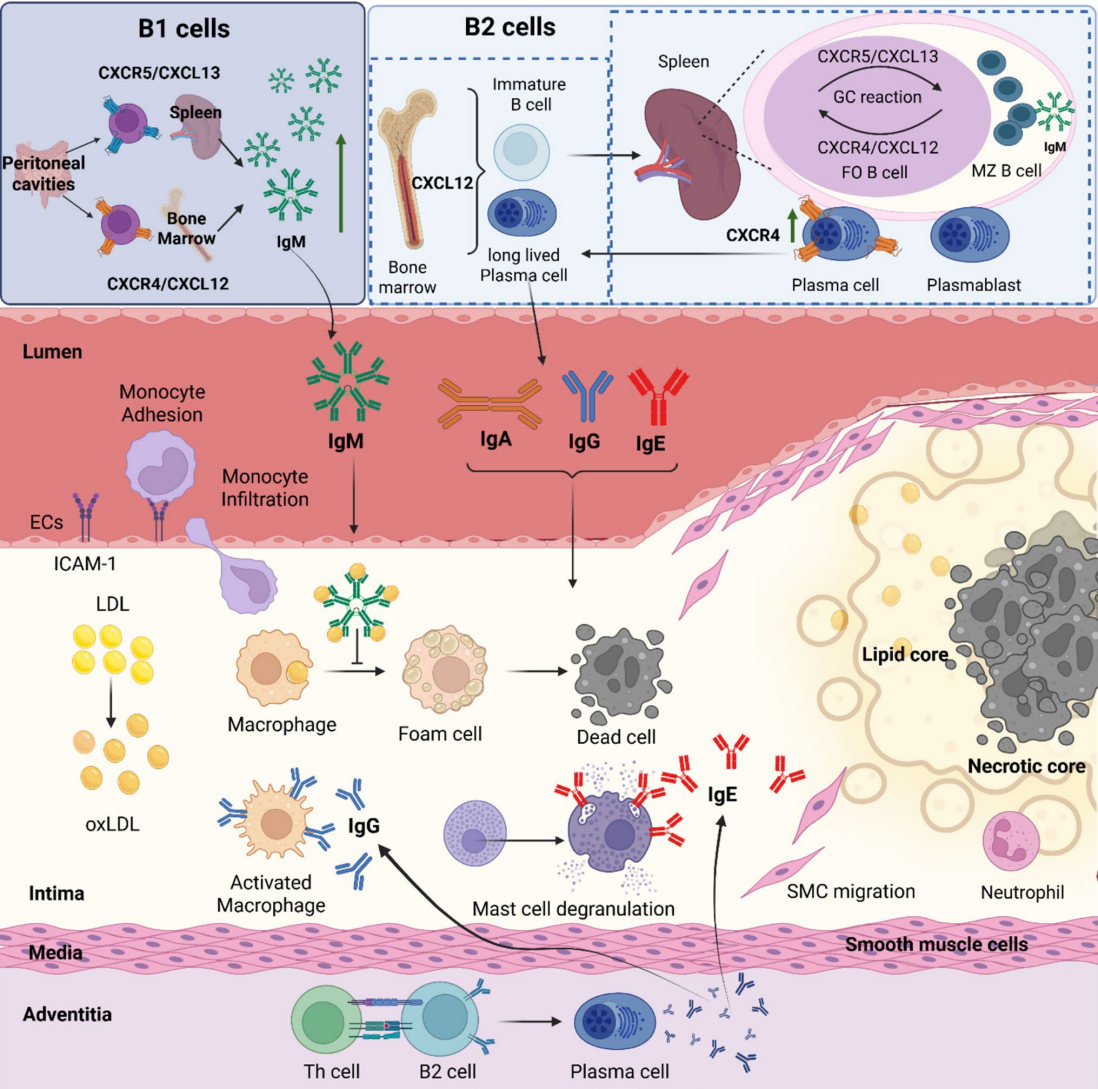
Role of chemokine (receptors) in B-cell immune responses in atherosclerosis. B1 cell migration to the peritoneal cavities and bone marrow is mediated via CXCR4/CXCL12 signaling and increased atheroprotective IgM production. On the other hand, B2 cells mature in the spleen, where the germinal-center (GC) reaction occurs. B2 cell maturation into plasma cells in the GC reaction is guided by a CXCR5/CXCL13 and CXCR4/CXCL12 chemokine gradient. Long-lived plasma cells migrate back to the bone marrow by increasing their surface CXCR4 and homing to the CXCL12-rich bone marrow, while plasmablasts reside in secondary lymphoid organs or tissue, where they will secrete different antibody subsets, such as IgA, IgGs and IgE. Circulating antibodies can enter the intimal layer of the vessel and activate various immune cells. In the context of atherosclerosis, monocytes adhere to activated endothelial cells (ECs) expressing ICAM molecules and infiltrate the arterial intima, where they differentiate into macrophages. These macrophages ingest oxidized low-density lipoproteins (oxLDL) and become foam cells, which ultimately triggers macrophage cell death contributing to the formation of a necrotic core. Antibodies produced by B cells play various roles in this process. For example, atheroprotective IgMs recognize modified lipids and prevent their uptake by macrophages and, therefore, prevent foam-cell formation. Atheroprogressive IgGs can bind to Fc-receptors on macrophages which leads to their activation and secretion of pro-inflammatory cytokines. Atheroprogressive IgE antibodies can bind to mast cells and thereby induce their degranulation and release of histamine. The figure was created in BioRender.com

The immune system also plays a crucial role in the pathogenesis of atherosclerosis, which is a common underlying cause of many CVD conditions. Among B2 cells, FO B cells maturing into plasma cells are mainly proatherogenic by producing pro-inflammatory antibodies that contribute to inflammation and atherosclerosis (Fig. 3) [2, 19, 67]. Germinal-center-derived IgG forms immune complexes that engage activating Fcγ receptors on lesional macrophages, augmenting cytokine production and plaque growth [19]. MZ and B1 cells on the other hand help prevent atherosclerosis development by producing large quantities of natural IgM antibodies directed towards oxidation-specific epitopes present on apoptotic cells and modified lipids[11, 90] Natural IgM from B-1/MZ B cells targets oxidation-specific epitopes (e.g., phosphorylcholine on oxLDL), opsonizes apoptotic bodies, limits foam-cell formation, and reduces necrotic cores; adoptive transfer and loss-of-function studies demonstrate causal protection (Fig. 3) [68, 69]. For example, Human CD24^hi^ marginal-zone-like B cells produce atheroprotective IgM to atherogenic antigens and associate with reduced vascular disease, underscoring translational relevance [86]. Finally, IgE can potentiate mast-cell degranulation and protease release, providing a humoral link to cap destabilization. Collagen architecture and microcalcifications, shaped by inflammatory milieus, in addition determine cap rupture mechanics, a context in which B-cell-driven effector pathways may modulate risk [60]. Furthermore, MZ B cells have been shown to limit excessive adaptive immune responses by follicular helper T cells to high-cholesterol conditions [81]. In addition, Bregs dampen inflammation, e.g., via IL-10 production [91], and may thus behave atheroprotective, although another study could not confirm a role of B-cell-derived IL-10 in atherosclerosis[92]. B2 cells are primarily located in the adventitia, whereas B1 cells are mainly found in the perivascular area, where they secrete antibodies (Fig. 3) [93, 103]. B2 cells can also be found in tertiary lymphoid organs (TLO) in the adventitia, forming at sites of chronic inflammation. TLOs are ectopic lymphoid structures, where GC reactions can occur [97]. However, single-cell and profiling studies refine the macrophage atlas underlying plaque instability, providing a scaffold to also better map B-cell/antibody–macrophage interactions which may also refine again location preferences [46].

Chemokines and their receptors play an important role during atherosclerosis development and are implicated in the recruitment, adhesion, activation, and differentiation of immune cells, as well as in phagocytosis [30, 31, 43]. In murine models, CCR6 + B1 cells are more frequently found in antibody-secreting sites, such as PVAT, spleen, and BM, compared to primary niches, like the peritoneal cavities. The absence of CCR6 (in *Apoe*^*−/−*^*Ccr6*^*−/−*^ mice) significantly reduces the number of IgM-secreting B1 cells in PVAT, highlighting the crucial role of CCR6 in recruiting B1 cells to this tissue. This recruitment is associated with local IgM production, which helps attenuate atherosclerosis. Despite reduced B1 cell numbers in PVAT, plasma IgM levels remain unchanged, suggesting a localized effect of IgM in atheroprotection [102]. In addition, the expansion of B1a cells in the PVAT is mediated by IL-5, which is locally produced through IL-33-induced activation of natural type-2 innate lymphoid cells (nILC2s) via the helix–loop–helix factor inhibitor of differentiation 3 (Id3) [87]. Furthermore, in 2007, a genome-wide association study (GWAS) revealed CXCL12 as an important gene involved in CVDs. Indeed, increased CXCL12 levels were associated with increased risk of heart failure and CAD severity [74, 88]. CXCL12 is proatherogenic due to its role in dyslipidaemia, angiogenesis, thrombus formation, plaque destabilization, neointima hyperplasia, and pro-inflammatory effects [29, 42, 72]. However, the role of the CXCL12/CXCR4/ACKR3 axis in atherosclerosis includes a large range of cell-type-specific biological effects [77]. Regarding B-cell in vivo, CXCR4 was shown to promote an IgM antibody response after T-cell-independent activation by the antigen NP-Ficoll [79]. In addition, CXCR4-deficiency on B cells resulted in loss of the B1 cell population in the peritoneal cavity, suggesting that CXCR4 is crucial for the homing and maintenance of an atheroprotective B1 pool [79]. Another study showed that CXCL12 is important for the migration and homing of both B1 and B2 cells in the peritoneal cavity, with antibody-mediated blocking of CXCL12 resulting in the local loss of both B-cell subsets [103].

A human cohort study found a positive association between CXCR4 expression on circulation B1 cells and plasma levels of IgM specific for malondialdehyde (MDA)-modified lipids. The same study reports an inverse correlation between the expression level of CXCR4 on B1 cells and coronary artery plaque burden and necrosis [108] and shows in *Apoe*^*−/−*^ mice with a B-cell-specific knockout of CXCR4 a decreased IgM production in the BM as well as a reduction in IgM plasma levels, associated with enhanced aortic atherosclerosis lesion size (Fig. 3). In line, B1-specific CXCR4 overexpression resulted in increased IgM and MDA-IgM levels in the plasma and promoted B1 cell migration in the BM [108]. Similar results were described in female B-cell-specific CXCR4-deficient *Apoe*^*−/−*^ mice, as the lack of CXCR4 decreased B1 cells in the BM, reduced plasma IgM, and led to increased aortic root and arch lesion sizes, while B2 cell numbers stayed unchanged [28]. Taken together, an atheroprotective role has been revealed for CXCR4 on B cells. On the other hand, macrophage migration-inhibitory factor (MIF), a non-cognate ligand of CXCR4 as well as of CXCR2 and the CD75–CD44 complex, mediates atherosclerosis in a B-cell-dependent manner [97]. Global *Mif*-deficiency impaired B-cell maturation in the BM and reduced the number of circulating B-cell numbers [97]. In line, MIF-producing DCs were found to be necessary for the survival of mature recirculating B cells in the BM [95]. In contrast to B-cell-specific CXCR4-deficiency[95], total *Mif*-deficiency was atheroprotective and associated with an increased atheroprotective autoantibody phenotype[97], suggesting that the impact of MIF on B-cell phenotype concerning atheroprogression is mediated via its other receptors, such as CXCR2[65], CD74[97] or ACKR3[3] but not CXCR4.

Elevated CXCL12 associates with coronary artery disease and heart-failure risk, yet CXCR4 signaling in B cells often appears atheroprotective—supporting retention and migration into bone-marrow and PVAT niches that favor natural IgM production.[29, 42, 77] Paradoxically, CXCR4, the main receptor for CXCL12, often exerts atheroprotective effects [28]. This raises the possibility that some atherogenic CXCL12 effects may be mediated through the alternative receptor ACKR3. ACKR3 binds CXCL12 with tenfold higher affinity than CXCR4 and acts as a chemokine scavenger and triggers distinct β-arrestin-mediated signaling pathways involved in cell survival, proliferation, and metabolism, processes highly relevant to cardiovascular pathology [50, 63, 88, 111]. In B cells, ACKR3 is enriched on specific subsets—marginal-zone B cells in mice and memory B cells/plasmablasts in humans—with dynamic regulation during differentiation and an inverse relationship with CXCR5 as cells transition from GC to periphery [58, 77, 89, 101]. Pharmacologic inhibition or genetic disruption of ACKR3 perturbs splenic marginal-zone organization and reduces MZ B-cell numbers; transfer of ACKR3-deficient MZ B cells impairs antigen delivery to FDCs and blunts T-independent IgM responses, indicating a non-redundant role in early humoral immunity [89, 109]. Together, by scavenging CXCL12 and shaping its gradients, ACKR3 can indirectly tune CXCR4 chemotaxis and positioning of B-cell subsets [32, 50, 63, 88]. Although direct evidence in atherosclerosis is currently lacking, ACKR3’s expression pattern and gradient-modulating capacity make it a plausible regulator of B-cell trafficking and antibody output in cardiovascular inflammation.

Overall and given these B-cell-centric mechanisms, chemokine-targeted interventions (Chapter 6) may ultimately benefit from strategies that preserve atheroprotective B1/MZ functions while limiting pathogenic humoral responses.

## Therapeutic approaches of chemokine targeting

Chemokines and their receptors are promising but underexploited therapeutic targets in CVD. Particularly, atherosclerosis, MI, and heart failure involve chronic inflammation, endothelial dysfunction, and leukocyte recruitment—all regulated by chemokine signaling. CCR2 is currently a leading target; blocking its ligand CCL2 via CCX140 reduces plaque inflammation in mice [15, 16]. CCR2 inhibition has also been tested in clinical trials, where it has been shown to reduce the inflammation marker high-sensitivity C-reactive protein (hsCRP) in patients with atherosclerotic risk and increased hsCRP [47]. In addition, it has been demonstrated to be nephroprotective in patients with diabetic nephropathy [27]. Although cardiovascular outcome trials are not yet available, carriers of a damaging CCR2 variant were shown to be at lower risk of MI and CAD [45]. Furthermore, CXCR2 antagonists (e.g., AZD5069, navarixin) block neutrophil chemotaxis and may reduce myocardial damage in ischemia–reperfusion injury and heart failure. Preclinical results show smaller infarcts and improved cardiac function; though trials have focused on respiratory inflammation, cardiovascular applications seem justified [55, 113]. For the CXCL12–CXCR4 axis, the CXCR4 antagonist mavorixafor, which mobilizes stem and endothelial progenitor cells, is under investigation for harnessing this reparative potential; however, only in patients with WHIM syndrome, a rare immunodeficiency caused by gain-of-function of CXCR4 gene [7].

Several T-cell-related chemokine receptors, such as CCR1, CCR4, CCR9, CCR5, and CX3CR1, also show therapeutic potential [70]. CCR1 antagonists, such as CCX354-C and BX471, showed safety and modest efficacy in rheumatoid arthritis by dampening T-cell-driven inflammation; such effects may translate to reduced vascular inflammation in CVD [84, 105]. CCR4 targeted by mogamulizumab depletes CCR4^+^ T cells to treat T-cell lymphomas [59]. CCR9 antagonized by CCX282-B (Vercirnon) in Crohn’s disease reduced pathogenic T cell trafficking [37]. In addition, CCR5 inhibition via Maraviroc has demonstrated vascular anti-inflammatory effects in HIV patients and holds promise for broader CVD indications [23, 39]. Collectively, these chemokine axes highlight the significance of T-cell-mediated immunity in cardiovascular inflammation and serve as key targets for future therapeutic development.

Despite significant advances in targeting chemokine pathways implicated in monocyte, neutrophil, and T cell recruitment in CVD, therapeutic strategies directly addressing B-cell-mediated immune mechanisms remain notably underdeveloped. This is a critical gap given the growing recognition of B cells and their associated chemokine receptors in orchestrating tertiary lymphoid structures and sustaining chronic vascular inflammation, especially in autoimmune and inflammatory forms of atherosclerosis. Murine studies have shown that naive follicular B cells, guided by CXCR5, infiltrate the heart following ischemic injury and contribute to inflammation and dysfunction [1]. In humans, lower CXCR5 expression on naive B cells has been observed in responders to cardiac resynchronization therapy (CRT), suggesting that reduced B-cell infiltration into the myocardium may enhance therapeutic response by limiting maladaptive inflammation and fibrosis [10]. In addition, emerging therapeutic strategies targeting the CXCL13–CXCR5 axis are under investigation, particularly in autoimmune diseases, with growing translational relevance to cardiovascular inflammation [99]. The monoclonal antibody MAb 5261, designed to neutralize CXCL13, has shown promising preclinical efficacy in models of collagen-induced arthritis and experimental autoimmune encephalomyelitis by selectively disrupting the recruitment of CXCR5-expressing B cells and Tfh ells into inflamed tissues, thereby reducing local immune-cell accumulation and cytokine production without triggering anti-drug immune responses [99]. Another agent, PF-06835375, a humanized monoclonal antibody against CXCR5, has advanced to Phase I clinical trials in systemic lupus erythematosus (SLE) and rheumatoid arthritis. This agent demonstrated favorable pharmacokinetics and safety, and importantly, modulated gene expression signatures associated with B cells and Tfh cells, supporting effective receptor engagement and downstream immune modulation. Although efficacy data are pending, these results validate the biological activity of CXCR5 blockade in humans [25]. While no therapies targeting CXCL13 or CXCR5 have yet been evaluated clinically in CVD, their demonstrated role in driving tertiary lymphoid structure formation, B-cell–Tfh-cell interactions, and autoantibody production in vascular tissues suggests significant potential for therapeutic translation in atherosclerosis, myocarditis, and autoimmune-related CVD [70]. Another (translational) example is anti-CD20-mediated B-cell depletion in acute MI. In this dose-escalation *Rituximab in patients with acute ST-elevation myocardial infarction* (RITA-MI) phase 1/2a study, a single rituximab infusion (200–1000 mg) administered in the acute ST-elevation MI (STEMI) setting was safe, induced rapid (~ 96%) depletion of circulating CD19⁺ B cells within minutes, and did not lower total IgG/IgM/IgA levels over 6 months, while repopulation was dose-dependent [114]. Hence, an ongoing RITA-MI 2 phase IIb randomized, double-blind, placebo-controlled trial (NCT05211401) is now testing whether a single 1000 mg dose (vs. placebo) improves 6-month left ventricular ejection fraction by cardiac magnetic resonance after anterior STEMI; a vascular-inflammation sub-study is planned. Mechanistically this B-cell depletion in MI targets mature B-cell-driven CCL7 production and Ly6C^hi^ monocyte mobilization, processes linked to worse remodeling in mice and associated with higher CCL7/BAFF in patients, suggesting a plausible axis by which rituximab could modulate post-MI inflammation [115]. Nevertheless, because natural IgM can be cardioprotective, timing and dose are likely critical to preserve protective humoral functions while dampening pathogenic responses. Yet, combining B-cell-directed approaches with chemokine-axis modulation (e.g., CXCL12–CXCR4–ACKR3) could help balance protective vs. pathogenic humoral programs during remodeling and warrants stratified testing.

Collectively, while no chemokine-targeted therapy is currently explicitly approved for cardiovascular indications, the preclinical and translational data are compelling.

## Conclusion and outlook

This review highlights the diverse roles of chemokines and chemokine receptors in mediating B-cell functions in CVD, with particular focus on MI, stroke, and atherosclerosis. B cells influence disease pathology through chemokine–receptor interactions that regulate their recruitment, positioning, and activation, ultimately shaping inflammatory responses, humoral immunity, and tissue remodeling. Key receptors such as CXCR4, CXCR5, CCR2, and ACKR3 are crucial for guiding B-cell migration and splenic organization, thereby affecting antibody production and immune regulation.

The chemokine system is characterized by receptor promiscuity, ligand redundancy, and dynamic receptor interactions, including homo- and heterodimerization, making it challenging to dissect precise biological functions. This complexity underscores the need to investigate chemokine receptor signaling in a cell- and disease-specific context. Although systemic clinical trials targeting chemokine receptors have shown promising early outcomes—such as reduced inflammatory markers and improved vascular function—long-term efficacy and safety remain to be established, particularly given the risk of off-target effects.

However, chemokine receptor-directed manipulation of B cells may represent a promising avenue. Enhancing the recruitment of atheroprotective B1 cells or limiting pro-inflammatory B2 responses through selective chemokine targeting could stabilize plaques and mitigate disease progression. In particular, ACKR3’s role in regulating CXCL12 bioavailability and modulating CXCR4-mediated chemotaxis positions it as an intriguing target to fine-tune B-cell activity in atherosclerosis.

Nevertheless, most mechanistic insights into chemokine–B-cell interactions stem from murine models, and interspecies differences remain a significant limitation. For example, MZ and FO B cells display distinct roles in murine vs. human atherosclerosis, and murine MZ B cells rely strongly on ACKR3 for positioning and maturation, a dependency not yet confirmed in humans. Despite these caveats, conserved functions—such as CXCR4/CXCL12-mediated B-cell migration and IgM production—support cautious optimism for translational potential.[17, 58, 109] Moving forward, clarifying the role of ACKR3 in human B-cell-mediated immunity, alongside careful validation of murine findings in human cells and patient-derived tissues, will be essential.

Moreover, future strategies in general will likely rely on precise phenotyping, biomarker-guided patient selection, and optimized timing of intervention to balance immune suppression with reparative capacity. Emerging cell-specific approaches, including antibody-coated nanoparticles, offer further potential to improve targeted delivery while minimizing systemic side effects.[23] Importantly, the CANTOS trial has already demonstrated that targeting inflammation can reduce cardiovascular events, validating immunomodulation as a therapeutic strategy in CVD.[51]

By addressing these open questions, future research can more precisely define how chemokine receptor-mediated immune cell and particularly B-cell responses contribute to CVD pathogenesis and repair.

## Data Availability

No new datasets were generated or analyzed for this review article. All figures are original and were created by the authors using BioRender.

## Abbreviations

ACKR: Atypical chemokine receptor
BCR: B-cell receptor
BDNF: Brain-derived neurotrophic factor
Blimp-1: B lymphocyte-induced maturation protein 1
BM: Bone marrow
Breg: Regulatory B cell
CAD: Coronary artery disease
CD40L: CD40 ligand
CSR: Class-switch recombination
CVD: Cardiovascular disease
DAMPs: Damage-Associated Molecular Patterns
DC: Dendritic cell
DRYLAIV: Asp–Arg–Tyr–Leu–Ala–Ile–Val
FDC: Follicular dendritic cell
FO: Follicular
GC: Germinal center
GWAS: Genome wide association study
HSC: Hematopoietic stem cell
LN: Lymph node
MDA: Malondialdehyde
MI: Myocardial infarction
MIF: Macrophage migration-inhibitory factor
MAPK: Mitogen-activated protein kinase
MI/RI: Myocardial ischemia–reperfusion injury
MZ: Marginal zone
NGF: Nerve growth factor
oxLDL: Oxidized low-density lipoproteins
PAMPs: Pathogen-associated molecular patterns
PALS: Periarteriolar lymphoid sheaths
PVAT: Perivascular adipose tissue
T_H_ cell: T-helper cell
TLO: Tertiary lymphoid organ
TLR: Toll-like receptor
7TM: 7 Transmembrane
S1P: Sphingosine-1-phosphate
S1P1: Sphingosine-1-phosphate receptor
SHM: Somatic hypermutation
WHO: World health organization
